# The complete mitochondrial genome sequence of Yarkand hare (*Lepus yarkandensis*)

**DOI:** 10.1080/23802359.2019.1681321

**Published:** 2019-10-23

**Authors:** Ya-Lin Huang, Yun-Xia Chen, Hai-Tao Guo, Yan-Hong Xu, Hong-Yi Liu, Da-Wei Liu

**Affiliations:** a Key Laboratory of State Forest and Grassland Administration on Wildlife Evidence Technology, Nanjing Forest-police College, Nanjing, China;; b Criminal Science and Technology Faculty of Nanjing Forest Police College, Nanjing, China;; c College of Biology and the Environment, Nanjing Forestry University, Nanjing, China

**Keywords:** *Lepus yarkandensis*, Yarkand hare, mitogenome

## Abstract

We first reported the mitochondrial genome of *Lepus yarkandensis*. The mitogenome of *L. yarkandensis* contains 17,011 base pairs. The overall base composition of complete mitogenome is 28.13% A, 27.67% T, 22.02% C, and 22.17% G, with 44.20% of the GC content. All genes exhibit the typical mitochondrial gene arrangement and transcribing directions. Phylogenetic analysis of 9 *Lepus* species was performed based on the sequence of cytochrome b gene using the Maximum Likelihood method in MEGA 7.0. The results suggested that *L. yarkandensis* is closely related to *Lepus timidus*. The results are helpful to future studies on molecular evolution, population genetics, and wildlife protection of *L. yarkandensis*.

Yarkand hare (*Lepus yarkandensis*) is a unique species in China. It is only distributed in the scattered oasis and desert area around Tarim Basin, Taklimakan Desert in Xinjiang Uygur Autonomous Region (Gao 1983). Due to the barrier of the desert, the *L. yarkandensis* population is naturally divided into small isolated groups that cannot communicate with each other. Meanwhile, as the development of local economy and oil exploitation industry as well as illegal hunting, *L. yarkandensis* population is facing a sharp decline and it has been listed as an Endangered wildlife since 1989 by Chinese government. Compared with other protected species, *L. yarkandensis* received less attention. Hence little information on the complete mitogenome of *L. yarkandensis* was available. In this study, we characterized the complete mitogenome of *L. yarkandensis* by using next-generation sequencing techniques.

Tissue samples of *L. yarkandensis* were collected from Kuqa County (41°68′N, 82°97′E) in Aksu region in January 2019 and after sampling, the specimens (NJFPC-201930) were stored in the animal specimens museum of Nanjing Forest-police College. mtDNA was isolated (Chen et al. 2011) and 1 μg of purified mtDNA was fragmented and used to construct short-insert libraries (insert size 430 bp) according to the manufacturer’s instructions (Illumina, San Diego, USA), then sequenced on the Illumina Hiseq 4000 (Borgstrom et al. 2011). Prior to assembly, raw reads were filtered at first. Then, the filtered reads were assembled into contigs using SOAPdenovo2.04 (Luo et al. 2012). The mitochondrial genes were annotated using homology alignments and denovo prediction and the EVidenceModeler v1.1.1 (Haas et al. 2008) was used to integrate gene set. A whole mitochondrial genome Blast (Altschul et al. 1990) search was performed against 5 databases.

The length of the complete mitogenome of *L. yarkandensis* is 17,011 bp (GenBank accession: MN450151). The complete mitogenome is relatively AT-rich (that is 55.8 vs. 44.20% of the GC content) with the following nucleotide compositions: 28.13% A, 27.67% T, 22.02% C, and 22.17% G. The mitogenome consists of 22 transfer RNA genes, 13 protein-coding genes, 2 ribosomal RNA genes (i.e. one rrnL and one rrnS) and one control regions. Most of the genes are encoded on the heavy (H) strand, except for those encoding nad6 and eight tRNAs. All genes follow the typical mitochondrial gene arrangement and transcribing directions, which is identical to most *Lepus* species (Yu et al. 2015; Ding et al. 2016; Giannoulis et al. 2018).

Phylogenetic analysis of 9 *Lepus* species was performed based on the complete mitogenome of *L. yarkandensis* and the nucleotide sequences of cytochrome b (Cyt b) of other 8 *Lepus* species, using the maximum likelihood method based on the Tamura-Nei model in MEGA version 7.0 (Tamura and Nei 1993; Kumar et al. 2016). Phylogenetic tree showed that the *L. yarkandensis* is closely related to *Lepus timidus* (Figure 1). The genome information obtained here could contribute to future studies on molecular evolution and wildlife protection in *L. yarkandensis*.

**Figure 1.**
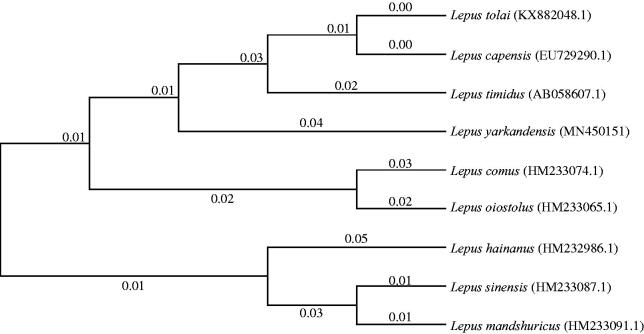
Phylogeny of 9 *Lepus* species based on the complete mitogenome of *L. yarkandensis* and the nucleotide sequences of cytochrome b of other 8 *Lepus* species using maximum likelihood method.
